# The transition state structure for coupled binding and folding of disordered protein domains

**DOI:** 10.1038/srep02076

**Published:** 2013-06-25

**Authors:** Jakob Dogan, Xin Mu, Åke Engström, Per Jemth

**Affiliations:** 1 Department of Medical Biochemistry and Microbiology, Uppsala University, BMC Box 582, SE-75123 Uppsala, Sweden

## Abstract

Intrinsically disordered proteins are abundant in the eukaryotic proteome, and they are implicated in a range of different diseases. However, there is a paucity of experimental data on molecular details of the coupled binding and folding of such proteins. Two interacting and relatively well studied disordered protein domains are the activation domain from the p160 transcriptional co-activator ACTR and the nuclear co-activator binding domain (NCBD) of CREB binding protein. We have analyzed the transition state for their coupled binding and folding by protein engineering and kinetic experiments (Φ-value analysis) and found that it involves weak native interactions between the N-terminal helices of ACTR and NCBD, but is otherwise "disordered-like". Most native hydrophobic interactions in the interface between the two domains form later, after the rate-limiting barrier for association. Linear free energy relationships suggest a cooperative formation of native interactions, reminiscent of the nucleation-condensation mechanism in protein folding.

Intrinsically disordered proteins (IDPs) and disordered regions of proteins are crucial for central cellular processes such as receptor signalling, cell-cycle control, and transcription^1^,^2^,^3^,^4^,^5^. It is believed that the flexibility of IDPs gives a functional advantage, for example, it enables binding of the polypeptide in an extended conformation with a larger interface area per residue than globular proteins^6^ and many specific contacts. The flexibility is also likely the reason that IDPs often can evolve to interact with multiple partners like, for example, p53^7^ and NCBD^8^,^9^,^10^,^11^ (see below). However, experimental data on details of the binding reaction mechanisms of IDPs are scarce and many of the hypotheses as to why disorder is beneficial remain untested by experiment.

Two disordered domains that participate in the formation of a multicomponent protein assembly that is involved in the activation and regulation of gene expression^12^,^13^ are the activation domain from the p160 transcriptional co-activator for thyroid hormone and retinoid receptors (ACTR) and the nuclear co-activator binding domain (NCBD) of CREB binding protein (CBP). These protein domains and their interaction have been characterized in detail by NMR^8^,^14^,^15^, SAXS^14^,^16^ and stopped-flow spectroscopy^17^. ACTR is highly disordered with some degree of secondary structure in the free state^15^,^16^, while NCBD has molten globule characteristics with a small hydrophobic core and low stability^14^,^15^,^18^,^19^. The bimolecular complex of the two domains displays a well-defined tertiary structure^8^ (Fig. 1) and is a classical example of coupled binding and folding of IDPs.

To shed light on the molecular details of the coupled binding and folding reaction of ACTR and NCBD we have here used protein engineering in conjunction with kinetic experiments (Φ value analysis^20^,^21^) to determine the structure of the transition state in terms of formation of native hydrophobic tertiary contacts in the binding interface. Our study, which is the first one reported on a system, in which one of the components is completely disordered (ACTR) and the other one has molten globule characteristics (NCBD), demonstrates that very few native interactions are in the process of forming in the transition state for binding. However, a nucleus with partial formation of native hydrophobic interactions was found to be present between the N-terminal helices of both IDP domains.

## Results

### Design of site-directed mutants

In a previous study we characterized the kinetic binding reaction of ACTR and NCBD using engineered Trp residues as fluorescent probes^17^. In the current study we made ten deletion mutations (purportedly non-disruptive side chain truncations) at hydrophobic residues in ACTR_WT_ and an additional ten in a pseudo wild type of NCBD (NCBD_Y2108W_). The mutations were made in the binding interface between the two domains based on a published NMR structure of the complex^8^. ACTR_WT_ is highly disordered in solution^8^,^15^ and mutations are not expected to disrupt any hydrophobic core. NCBD, on the other hand, displays significant residual structure as shown by NMR and circular dichroism (CD)^11^,^14^,^15^,^19^. Far-UV CD was used to assess the effect of the mutations on the secondary structure of NCBD (Supplementary Fig. S1). Six of the mutants displayed a CD spectrum identical to that of NCBD_Y2108W_, whereas four appeared to have lost some α-helical structure. Trimethylamine *N*-oxide (TMAO) is known to shift the equilibrium towards the folded state^22^ and 0.7 M partially or fully restored the CD spectra for these four mutants (Supplementary Fig. S1).

### Binding kinetics of mutants of ACTR and NCBD

**Figure 1:**
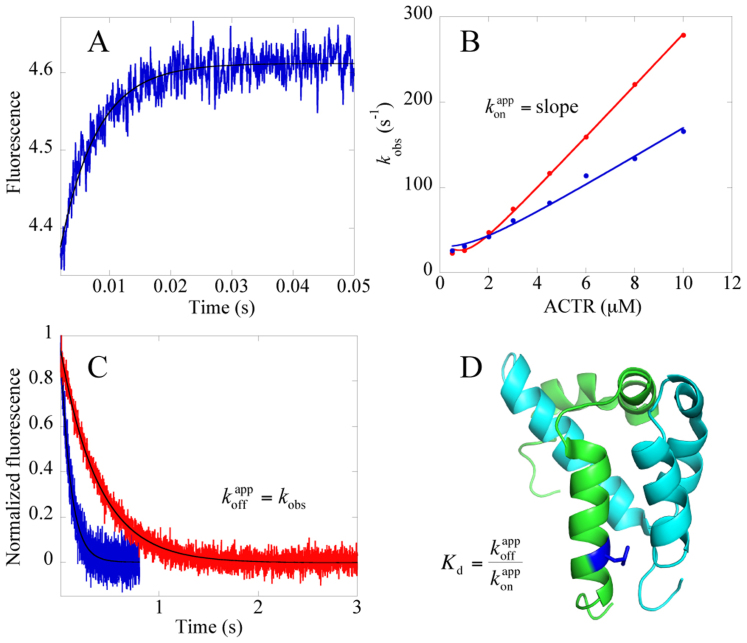
The experimental system. (A) Example of an experimental binding trace for NCBD_Y2108W_ and an ACTR mutant, L1048A. Shown here is the main (fast) phase and protein concentrations are 1 μM NCBD_Y2108W_ and 10 μM L1048A-ACTR. (B) *k*_obs_ values for the main phase obtained from binding traces (such as in panel A) plotted versus [ACTR] to obtain association rate constants, *k*_on_^app^ by fitting the data to the general equation for the reversible association of two molecules under second order conditions^46^ (Eq. 4). At high concentrations of ACTR the equation approaches a linear function with a slope equal to *k*_on_. Red, NCBD_Y2108W_ and ACTR_WT_; blue, NCBD_Y2108W_ and ACTR_L1048A_. (C) Dissociation rate constants, *k*_off_^app^, were determined in displacement experiments, in which NCBD_WT_ was used to compete out NCBD_Y2108W_ from the complex. The solid line is a fit to a single exponential equation. At high concentration of NCBD_WT_ the dissociation of NCBD_Y2108W_/ACTR is virtually irreversible and *k*_obs_ from the curve fitting is equal to *k*_off_^app^. Red, NCBD_Y2108W_/ACTR_WT_; blue, NCBD_Y2108W_/ACTR_L1048A_. (D) Structure of the complex between ACTR (green) and NCBD (cyan), with L1048 highlighted in dark blue. The figure was generated using PyMol (The PyMol Molecular Graphics System, Version 1.3 Schrödinger, LLC).

The binding kinetics of the ten ACTR mutants was measured with NCBD_Y2108W_ and the kinetics of the ten NCBD_Y2108W_ mutants measured with ACTR_WT_, using the stopped-flow technique (Fig. 1 and Supplementary Table S1, Fig. S2 and Fig. S3). Two of the mutations (L1064A and L1071A in ACTR) were highly destabilizing for the bimolecular complex and their kinetics were measured in the presence of 0.7 M TMAO to reduce the observed rate constants (*k*_obs_) to a magnitude accessible by the stopped-flow instrument. Likewise, the four mutants with altered CD spectra were measured in the presence of 0.7 M TMAO to avoid ground state effects on the observed kinetics. Two more mutants, L2090A NCBD and L1052A ACTR, were also purified but did not yield reliable kinetics data, due to elevated *k*_obs_ values, even in the presence of TMAO.

The binding kinetics of almost all mutants was biphasic, with a fast phase, which was linear with increasing concentration of ACTR and a slow phase, which appeared rather constant throughout the measured concentration interval. Similar kinetics was observed previously for NCBD_Y2108W_ and ACTR_WT_, and a detailed analysis has been published^17^ (see also text in Supplementary Information for further discussion on the slow phase). From the fast phase we extracted the apparent association rate constant (*k*_on_^app^) as the slope of the observed rate constant *k*_obs_ versus ACTR concentration (Fig. 1, Supplementary Table S1, Fig. S2). The apparent dissociation rate constant (*k*_off_^app^) for mutant ACTR/NCBD complexes was determined in separate displacement experiments (Fig. 1, Supplementary Table S1) as detailed in the Materials section.

**Figure 2:**
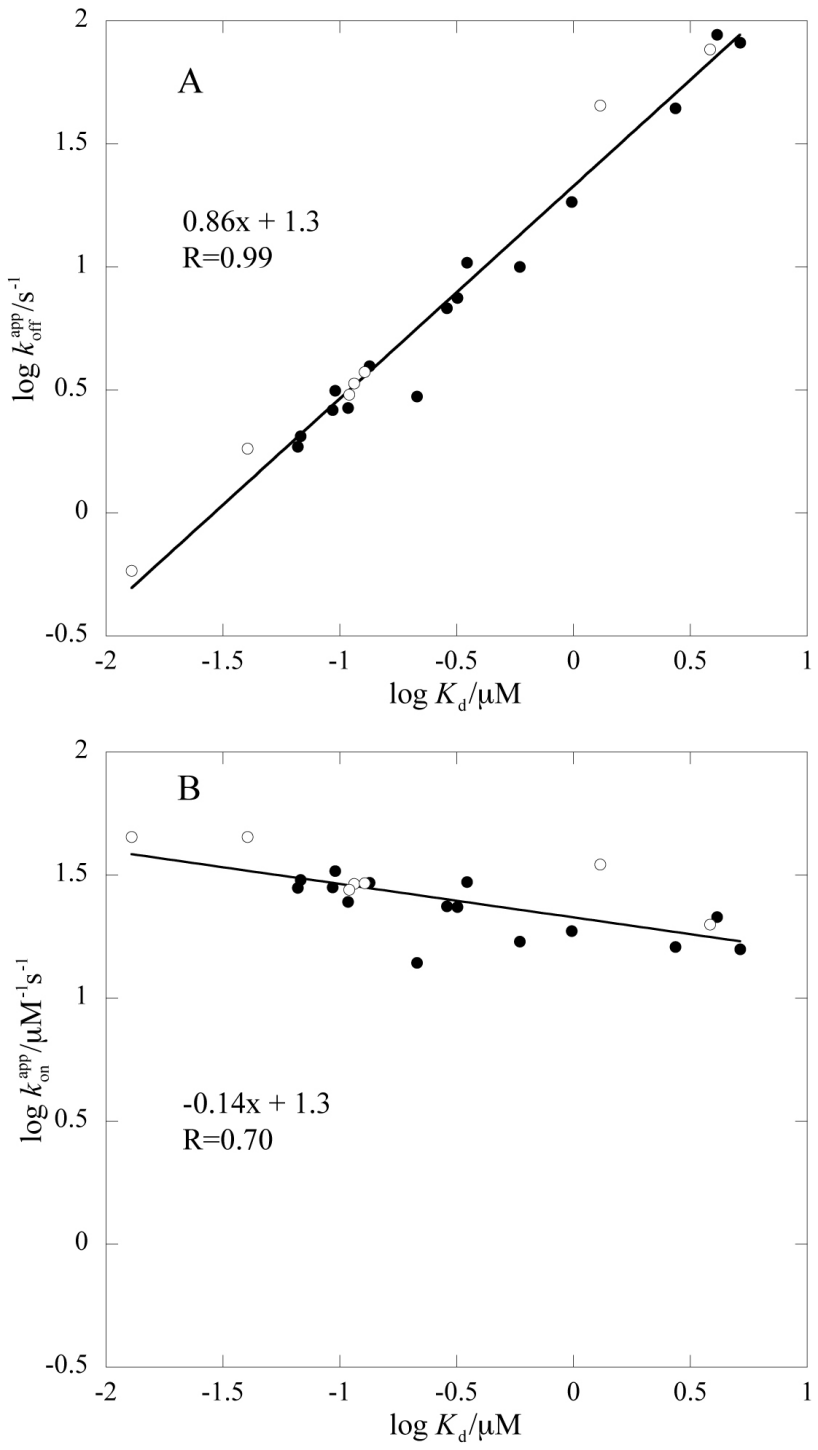
Linear free energy relationships of rate and equilibrium binding constants upon mutation. (A) log *k*_off_^app^ versus log *K*_d_ and (B) log *k*_on_^app^ versus log *K*_d_. Solid circles represent data that were obtained in 20 mM phosphate (pH = 7.4), 150 mM NaCl, whereas constants that were obtained in the same buffer but supplemented with 0.7 M TMAO are shown as open circles.

In the experiments with TMAO, an additional phase of intermediate magnitude was clearly visible for one mutant, L2067A (*k*_obs_~19 s^−1^). Interestingly, a similar phase was previously detected for a double mutant involving a buried salt bridge, but also for the wild type at high ionic strength^17^. Careful analyses of binding traces for NCBD_Y2108W_ and wild-type ACTR (i.e., our wild type pair) in 0.7 M TMAO revealed that a similar intermediate phase could be fitted to the data. This result may be explained by an induced-fit scenario, in which the population of an intermediate is promoted by TMAO, high salt, and certain mutations. Thus, TMAO and salt may be used to tune the shape of the energy landscape for coupled binding and folding for ACTR/NCBD. However, while the presence of TMAO may shift the distribution of populations of free ACTR and NCBD, and the height of energetic barriers, it is unlikely to affect the transition state structure and overall mechanism as shown by the linear free energy diagrams (Brønsted plot), in which data collected in presence and absence of TMAO fall on the same line (Fig. 2 and Supplementary Fig. S4B).

For three out of the four mutants, TMAO did not restore the magnitude of the CD signal completely, but they are close, having 90%, 88%, and 81% of the wild-type CD signal at 222 nm. However, as judged from Fig. 2, these small differences are not enough to result in a significant deviation from the overall linear trend in the Brønsted plot. Thus, for these mutants as well as the other ones measured in the presence of TMAO, the data report on the same transition state.

### Structural details of the interface between ACTR and NCBD

The 3D structure of the complex between ACTR and NCBD^8^ shows that the leucine rich binding interface of NCBD/ACTR is well packed and contains specific hydrophobic interactions. Accordingly, several of the conservative deletion mutations resulted in significantly reduced binding affinities (Table 1 and Supplementary Table S1), in particular those from Leu to Ala in LXXLL/LLXXL motifs. These results agree well with previous mutational studies of the interaction between NCBD and ACTR^19^ or TIF2, an ACTR homolog^23^, as well as other NCBD binding proteins^24^,^25^. On the other end, the mutations V2109A NCBD and V1077A ACTR were not destabilizing but displayed even lower *k*_off_^app^ and *K*_d_ values than the wild type (Supplementary Table S1). Both of these mutated residues are positioned at the C-terminal helices of the respective domain, and as shown in the NCBD/ACTR structure^8^ they also interact with each other. While the thermodynamic origin of these changes is not clear, it has previously been shown that the C-terminal part of helix three in unbound NCBD displays significant fast backbone dynamics^15^ compared to the rest of NCBD, and the region in bound ACTR that forms the C-terminal helix, has very little helical content in unbound ACTR^15^,^16^. One may speculate that the disorder that is present in the respective helix is modulated by mutation, for example through stabilization of the helix, such that a slightly higher affinity is obtained, compared to the wild type.

### Calculation of Φ_binding_ values

**Figure 3:**
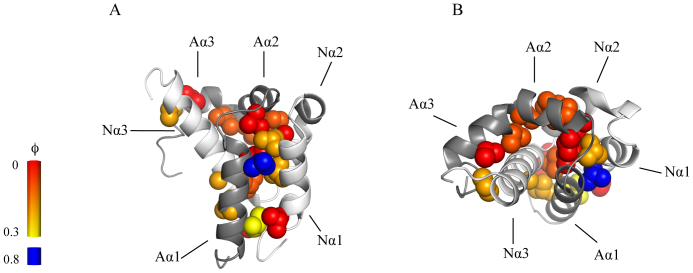
A structural model of the transition state for coupled binding and folding. Φ_binding _values from Table 1 and 2 mapped onto the structure of the complex between ACTR and NCBD. ACTR is shown in dark grey and NCBD in light grey. Residues are colour coded by the magnitude of their Φ_binding _values, with a gradient ranging from 0 (red) to 0.3 (yellow). ACTR_L1055A_, which has the highest Φ_binding _value, is blue colored. The negative Φ_binding _values were close to zero and are colored red. Panel *A* and *B* show the same structure from two different angles. Images were generated using PyMol (The PyMol Molecular Graphics System, Version 1.3 Schrödinger, LLC).

Linear free energy relationships clearly demonstrate that the effect of the mutations is largely in the dissociation rate constant (Fig. 2A). However, a few mutations affected the association rate constant (Supplementary Fig. S2), suggesting that some side-chains have begun to form native-like interactions in the rate limiting transition state for binding. In order to quantify the degree of native contact formation and thus get a picture of the structure of the transition state for the coupled binding and folding of ACTR/NCBD, we calculated Φ values for binding, Φ_binding_^21^,^26^ (Table 1, see Supplementary Information for a detailed discussion of the Φ_binding_ values in relation to *k*_on_^app^, *k*_off_^app^ and the slow phase for each plausible reaction scheme) and mapped the values onto the structure of the complex between the two proteins (Fig. 3). Φ_binding_ values were calculated by relating the change in free energy for the rate limiting barrier for binding (ΔΔ*G*_TS_, calculated from *k*_on_ values) with the total change in free energy for the binding reaction at equilibrium (ΔΔ*G*_Eq_, calculated from *K*_d_ values, which in turn were calculated from *k*_off_^app^ divided by *k*_on_^app^) (Equations 1–3, Supplementary Information). 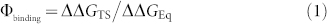

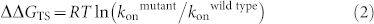

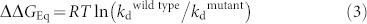
The wild type in the calculations refers to the interaction between NCBD_Y2108W_ and ACTR_WT_. A Φ_binding_ value of zero would imply that the mutated residue is not making any native interactions in the transition state of the binding reaction. A Φ_binding_ value of 1, on the other hand, implies that the residue makes a full native interaction in the transition state. Intermediate values are subject to a number of caveats^20^, but are usually interpreted as partial formation of native interactions in the transition state. We calculated a Φ_binding_ value if the absolute value of ΔΔ*G*_Eq_ > 0.16 kcal/mol. This may appear as a very low cut-off value, but is based on the very accurate determinations of *k*_on_^app^ and *k*_off_^app^. For most mutants, the largest source of error is the concentration determination of ACTR variants, which directly affects *k*_on_^app^ and which we estimate to about 5%. The Φ_binding_ values in Table 1 report on the transition state that is rate limiting for binding in 20 mM sodium phosphate (pH = 7.4), 150 mM NaCl. In the presence of TMAO, it is likely that the same transition state has been stabilized resulting in a higher *k*_on_^app^ value. An alternative explanation is that an earlier transition state becomes (partially) rate limiting. *k*_on_^app^ for NCBD_Y2108W_ and wild-type ACTR in presence of 0.7 M TMAO, was determined to be around 45 μM^−1^s^−1^, which was used to calculate Φ_binding_ values in Table 2.

### The structure of the transition state for coupled binding and folding

Following the guidelines of Fersht^20^, it is useful to categorize Φ values as low, intermediate and high. The low Φ_binding_ values (<0.2) at almost all positions show that most of the native hydrophobic interactions in the interface between ACTR and NCBD have not formed in the transition state for the binding reaction, suggesting that most of the initial encounter complex does not contain native-like hydrophobic interactions. However, a few ACTR mutations situated in helix 1 make direct interactions with NCBD and displayed intermediate or even high Φ_binding_ values. Although Φ_binding_ values for the NCBD mutants were low, two mutations in helix 1 gave Φ_binding_ values of 0.2, suggesting that the N-terminal helices of ACTR and NCBD form the initial native intermolecular contacts.

## Discussion

Protein engineering together with detailed kinetic analyses allowed us to determine a structural model of the rate-limiting transition state in the coupled binding and folding involving the IDP, ACTR, and the molten globule, NCBD (Fig. 3). In terms of hydrophobic interactions in the interface between the two disordered domains, the transition state for the initial association is more disordered-like than native-like. Overall, native interactions in the binding interface are formed and consolidated subsequent to the rate-limiting barrier for association. This is in agreement with previous studies on the kinetic binding mechanism of IDPs and disordered regions using NMR^27^, MD^28^ or stopped-flow spectroscopy^17^,^29^,^30^,^31^,^32^, in which data also suggest that native interactions form late on the reaction pathway. It is usually very difficult to prove multistep binding reactions and there are a number of possible binding mechanisms for the association of ACTR and NCBD. Importantly, our Φ_binding_ values report on the transition state for the productive binding pathway for either of the four mechanisms (Scheme 1–4 in Supplementary Fig. S5), which are overall consistent with the data set, as discussed in detail in Supplementary Information. Recent data on NCBD show that it exists in at least two different conformations in the free state^33^,^34^, which lends support to model 4 in Fig. S5. Thus, in their free states, there may be several different forms of ACTR and NCBD, with different degrees of structure and multiple pathways for binding, with external conditions determining the flux through each pathway^35^. However, the pathways converge after their initial association and ACTR and NCBD will experience a cooperative formation of native bonds as the barrier is crossed as shown by the linear free energy relationship (Fig. 2).

Looking at the transition state in more detail, a few native contacts have begun to form between the N-terminal helices of the respective protein in the transition state for binding. In fact, the linear free energy relationship (Fig. 2) is reminiscent of those obtained for proteins obeying the nucleation-condensation mechanism in protein folding^36^. Thus, following formation of a few native interactions during the crossing of the main rate-limiting barrier, the remaining native contacts form after the main barrier. Binding of IDPs often involves extended protein conformations with a binding surface containing hydrophilic as well as hydrophobic interactions. Interestingly, the region with highest Φ_binding_ values contains the LXXLL/LLXXL recognition motifs, where X stands for any amino acid. These motifs, in which any bulky hydrophobic residue can replace Leu, are known to mediate protein-protein interactions in transcriptional regulation^37^.

Furthermore, NMR and CD studies^15^,^16^ have shown that the region that constitutes helix one in bound ACTR in fact displays transient helix formation in the free state. Previous studies^38^,^39^,^40^,^41^ have emphasized the functional importance of preformed structural elements and our observation that such elements form the initial native contacts during the binding between ACTR and NCBD lends support to this hypothesis.

In our initial study on ACTR/NCBD^17^ we characterized a buried salt bridge by mutagenesis (R2104L in NCBD and D1068L in ACTR). It is clear that the effect on the association rate constant was large (~20 fold) while the *k*_off_^app^ was similar to that of the wild type proteins. This would imply a Φ_binding_ value of one, however, the R2104L mutation has clear effects on the ground state of NCBD^19^ and the two Leu residues that replaced the salt bridge will most probably contribute to binding by forming new hydrophobic interactions. Thus, while mutation of the salt bridge strongly affects *k*_on_^app^, suggesting that the salt bridge stabilizes the transition state for binding, we cannot estimate a Φ_binding_ value for this interaction. Previous studies on interactions involving IDPs^42^ or folded proteins^43^ have demonstrated that mutation of charged residues may affect the association rate constant such that high Φ_binding_ values are obtained. Thus, charged residues have a higher tendency than hydrophobic amino acids to form native-like interactions in the transition state for binding, due to long-range electrostatic effects^21^,^44^.

A recent molecular dynamics simulation study^38^ concluded that the end helices α1 and α3 in NCBD, which dominate the number of contacts it makes with ACTR in the complex, are most readily formed in the free state, having a conformation similar to the bound state. Here we show that α1 in NCBD forms weak native-like interactions with ACTR in the transition state, and two out of four mutations in α3 in NCBD has an intermediate Φ value (Table 1). It should be noted that these mutations involve hydrophobic substitutions, whereas α3 in NCBD also contains charged residues, which, as previously discussed, tend to adopt higher Φ values than hydrophobic positions^21^. Thus, it is possible that this region may make long-range native-like electrostatic interactions in the rate-limiting transition state.

In summary, we propose, based on available experimental data, the following scenario for the productive interaction between ACTR and NCBD. Helix one of the highly disordered ACTR forms transiently^15^,^16^, with folding and unfolding probably on the ns-μs timescale^45^. In a helix-like state it makes initial weak native-like interactions with helix one of NCBD that are rate limiting for the binding reaction. The initial association is also promoted by R2104 in NCBD and D1068 in ACTR^17^, which may steer the two protein domains into the correct orientation as the native salt bridge is formed. Formation of native hydrophobic contacts in the binding interface proceeds in a cooperative fashion following crossing of the rate-limiting transition state for association. It is likely that this mechanism is common for coupled binding and folding reactions of IDPs.

## Methods

### Protein expression and purification

Human NCBD and ACTR were expressed and purified as described previously^17^. Briefly, BL21(DE3) pLysS cells were used to express NCBD or ACTR in fusion with an N-terminal His-tagged lipoyl fusion protein with a thrombin site separating the lipoyl and NCBD or ACTR sequences. The fusion protein was first subjected to a Ni-sepharose fast flow (GE Healthcare) column purification step followed by thrombin cleavage, after which a second Ni-sepharose fast flow purification step was utilized to remove the lipoyl protein and other impurities. This was followed by reversed phase chromatography using a C-8 (Grace Davison Discovery) column. Mutations were generated by inverted PCR and mutants were expressed and purified as described for the wild-type proteins^17^. The identity of each mutant was verified with MALDI-TOF. The concentration of NCBD was determined by measuring the absorbance at 280 nm, whereas the concentration of ACTR, which lacks Tyr and Trp residues, was determined by measuring the absorbance at 205 nm and using an extinction coefficient of 31.9 mL mg^−1^ cm^−1^ obtained from amino acid analysis.

### Binding kinetics

Binding kinetics of NCBD/ACTR was measured using an upgraded SX-17MV stopped-flow spectrometer (Applied Photophysics, Leatherhead, U.K.) and monitoring Trp fluorescence^17^. Excitation was at 280 nm and a 320 nm long pass filter was used to monitor the emission. Measurements were done at 277 K in either 20 mM sodium phosphate (pH = 7.4), 150 mM NaCl, or the same buffer supplemented with 0.7 M TMAO. In order to obtain association rate constants (*k*_on_^app^) for NCBD_Y2108W_/ACTR^mutant^ and NCBD_Y2108W_^mutant^/ACTR_WT_, the concentration of ACTR was varied while keeping the concentration of NCBD constant at 1 μM. Observed rate constants were plotted versus [ACTR] and the data were fitted to the general equation for the reversible association of two molecules^46^ to determine *k*_on_^app^ (Fig. 1) (Eq. 4). 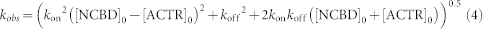
[NCBD]_0_ and [ACTR]_0_ are the total concentrations of the respective species. Note that the equation breaks down to a linear function under pseudo-first order conditions, that is when [ACTR]_0_ ≫ [NCBD]_0_.

Apparent dissociation rate constants were determined through displacement experiments, by mixing a pre-formed NCBD_Y2108W_/ACTR^mutant^ or NCBD_Y2108W_^mutant^/ACTR_WT_ complex (1.1–2.5 μM NCBD mixed with 1–2 μM ACTR) with an excess of [NCBD_WT_], such that ACTR is trapped in a spectroscopically silent complex. Higher concentration of mutant NCBD/ACTR complex was used for mutants with low affinity, to improve the amplitude of the kinetic trace. The observed rate constant at high [NCBD_WT_] is equal to *k*_off_^app^.

### Circular dichroism spectroscopy

CD spectra were recorded using a JASCO-810 spectrometer with a peltier temperature control system. Spectra were recorded between 260 nm and 200 nm, at 298 K or 277 K, and 20 mM phosphate (pH = 7.4), 150 mM NaCl, either with or without 0.7 M TMAO.

## Author Contributions

J.D. and P.J. planned and designed the experiments. J.D., X.M. and Å.E. performed the experiments. J.D. and P.J. analyzed data and wrote the paper.

## Supplementary Material

Supplementary Information Supplementary Information

## Figures and Tables

**Table 1:** Φ_binding_ values for the interaction between wild type and mutants of ACTR_WT_ and NCBD_Y2108W_, respectively, measured in 20 mM phosphate (pH = 7.4), 150 mM NaCl, and 277 K

	ACTR_WT_			NCBD_Y2108W_	
NCBD_Y2108W_ mutant	ΔΔ*G*_Eq_ kcal/mol	Φ_binding_	ACTR mutant	ΔΔ*G*_Eq_ kcal/mol	Φ_binding_
I2062V α1	0.20 ± 0.01	−0.12 ± 0.07	L1048A α1	1.02 ± 0.02	0.27 ± 0.02
V2086A α2	0.73 ± 0.02	−0.04 ± 0.02	L1049A α1	1.30 ± 0.02	0.17 ± 0.02
L2087A α2	2.2 ± 0.1	0.14 ± 0.03	L1055A α1	0.46 ± 0.05	0.85 ± 0.10
L2096A α3	1.9 ± 0.1	0.16 ± 0.03	L1056A α1	2.09 ± 0.1	0.07 ± 0.02
I2101V^a^ α3	0.01 ± 0.02	–	A1061G^a^ loop	0.08 ± 0.06	–
V2109A α3	−0.17 ± 0.02	0.22 ± 0.09	I1067V α2	0.62 ± 0.02	0.16 ± 0.02
			I1073V α3	0.68 ± 0.03	0.15 ± 0.05
			V1077A α3	−0.19 ± 0.02	−0.01 ± 0.13

aToo low ΔΔ*G*_Eq_ to calculate a reliable Φ_binding_ value.

**Table 2:** Φ_binding_ values for the interaction between wild type and mutants of ACTR_WT_ and NCBD_Y2108W_, respectively, measured in 20 mM phosphate (pH = 7.4), 150 mM NaCl, 0.7 M TMAO, and 277 K

	ACTR_WT_			NCBD_Y2108W_	
NCBD_Y2108W_ mutant	ΔΔ*G*_Eq_ kcal/mol	Φ_binding_	ACTR mutant	ΔΔ*G*_Eq_ kcal/mol	Φ_binding_
L2067A α1	0.63 ± 0.08	0.0 ± 0.1	L1064A α2	2.5 ± 0.1	0.06 ± 0.02
L2070A α1	1.2 ± 0.1	0.20 ± 0.04	L1071A loop/α2	3.1 ± 0.2	0.14 ± 0.03
L2074A α1	1.3 ± 0.1	0.19 ± 0.04			
A2099G α3	1.2 ± 0.1	0.23 ± 0.04			
