# Supplementary material for: The transition state structure for coupled binding and folding of disordered protein domains
Source: Sci Rep. 2013 Jun 25;3:2076. doi: 10.1038/srep02076 (PMC3691887; doi:10.1038/srep02076)
Supplement: Supplementary Information [file srep02076-s1.pdf]

Supplementary Information

The transition state structure for coupled binding and folding of disordered protein domains

Jakob Dogan[*,1], Xin Mu[1], Åke Engström[1] and Per Jemth[*,1]

[1]Department of Medical Biochemistry and Microbiology, Uppsala University, BMC Box 582, SE-75123 Uppsala, Sweden.

[*]Corresponding author. E-mail address: Jakob.Dogan@imbim.uu.se, Per.Jemth@imbim.uu.se, phone: +46-18-471 4557

Kinetic pathways and the calculated Φ value. The observed binding kinetics for ACTR and NCBD, with a fast linearly increasing phase and a slow phase (λ_{slow}), is compatible with several kinetic schemes (Supplementary Fig. S5). In addition to the binding experiments, we performed displacement experiments to obtain the apparent dissociation rate constant k_{off}^{app}. The parameter k_{off}^{app} together with its correlation with λ_{slow} (Supplementary Table S2, Fig. S4) put some constrains on the respective mechanism. It is a common misconception that Φ values cannot be determined for multi-state systems. In the present study we determine the structure of the transition state for the initial barrier for association. The meaning of the Φ values are discussed in detail below, for each scheme.

Scheme 1 (Supplementary Fig. S5) involves initial binding followed by a conformational change, which gives rise to the slow phase. In scheme 1, $\lambda_{slow} = k_2 + k_{-2}$

and $k_{off}^{app} = k_{-1}k_{-2}/(k_{-1}+k_{-2}+k_2)$. Thus, it can be shown that k_{off}^{app} cannot be greater than λ_{slow}, but experimentally, this is what we observe for all mutants (Supplementary Fig. S4). However, we cannot rule out this model since k_2 and k_{-2} may be of similar magnitude and what we observe as k_{off}^{app} may be the dissociation of the intermediate in Scheme 1, with an observed rate constant between k_{-1} and $k_{-1}k_{-2}/(k_{-2}+k_2)$, depending on the relative magnitudes of the microscopic rate constants. The correlation between λ_{slow} and k_{off}^{app} on mutation (Supplementary Fig. S4) is accounted for by this model since both parameters depend on k_{-2}. If this model applies, k_{-1} must be > k_2+k_{-2} and K_d^{app} will be close to the K_d for the first step. Thus, the $\Phi_{binding}$ values report on formation of native interactions in the first transition state, separating NCBD + ACTR and (NCBD:ACTR) in Scheme 1 (Supplementary Fig. S5). In addition, (NCBD:ACTR) and (NCBD:ACTR)* may be structurally similar since direct evidence of different bound conformations that are in exchange was not reported under the experimental conditions used in previous NMR studies[1].

Scheme 2 involves two parallel pathways, as suggested by molecular dynamics simulation[2]. This scheme would better account for the observation that $\lambda_{slow} < k_{off}^{app}$ values, since the latter now is approximately the sum of two k_{off}^{app} values, one for each path. Furthermore, scheme 2 predicts two λ_{slow} values, which are complex functions of all microscopic rate constants, accounting for the correlation between λ_{slow} and k_{off}^{app} upon mutation (Supplementary Fig. S4). Neither of the two slow phases would display a strong concentration dependence and they might well be very similar and, in practice, appear as one kinetic phase. Similarly to Scheme 1, $\Phi_{binding}$ values report on formation of native interactions in the first transition state, however, values will be the weighted mean of the two separate transition states for the initial

association, one leading to (NCBD:ACTR) and one to (NCBD:ACTR)*. Therefore, it is theoretically possible that intermediate $\Phi_{binding}$ values result from a high $\Phi_{binding}$ value for one path and a low one for the other path. The same caveat applies to protein folding Φ values[3].

Scheme 3 involves an off-pathway intermediate, or in other words, there are two distinct binding modes of ACTR and NCBD, with separate association and dissociation rate constants. The slow phase appears when the system approaches equilibrium following the initial binding event to both of the two conformations (NCBD:ACTR) or (NCBD:ACTR)*. In this scheme, $\lambda_{slow} = k_{-1}k_2/(k_1+k_2) + k_{-2}k_1/(k_1+k_2)$. This model fits nicely with the observation that k_{off}^{app} is greater than λ_{slow} for all mutants (Supplementary Fig. S4). However, scheme 3 predicts double exponential dissociation kinetics, with one phase equal to k_{-1} and the other equal to k_{-2}, which we only observe for two mutants, A1061G ACTR and I1073V ACTR. For these mutants, the amplitudes of the two dissociation phases displayed opposite signs, which is not expected. Another problem with this model is that any reasonable rate constants result in a large fraction (10-35%) of the minor bound species (NCBD:ACTR)* at equilibrium. Such a large fraction of the minor bound species should have been detected in earlier NMR experiments,[1,4] since the structural difference between (NCBD:ACTR) and (NCBD:ACTR)* does not allow the two bound conformations to interconvert without dissociating the complex. In scheme 3, $\Phi_{binding}$ values would reflect a weighted mean of the two distinct barriers separating free ACTR and NCBD from (NCBD:ACTR) or (NCBD:ACTR)*, respectively. In practice, since only one bound species is detected in NMR experiments, the association rate constant k_1 for the productive pathway must be 10-fold or so higher

than that of the non-productive one. The apparent K_d ($k_{off}^{app}/k_{on}^{app}$) will be close to the K_d for the binding of the major species and, hence, the $\Phi_{binding}$ values would report on the productive pathway.

Scheme 4, finally, depicts a scenario, in which NCBD exists in two distinct conformations in the free state. Two recent publications lend support to this model by demonstrating the presence of distinct NCBD conformations using NMR[5] and single molecule nanopore experiments,[6] respectively. In this model, the slow phase is virtually equal to the dissociation rate constant (k_{-2} in Scheme 4) for the minor species NCBD*. The k_{off}^{app} is equal to the dissociation rate constant (k_{-1}) for the major species. The correlation between λ_{slow} and k_{off}^{app} on mutation (Supplementary Fig. S4) is however not obvious, since they are depending on different microscopic rate constants in scheme 4. The correlation may, however, be explained by a similar relative loss of binding energy on mutation for the two complexes, (NCBD:ACTR) or (NCBD:ACTR)*. In scheme 4, k_{on}^{app} can be approximated as the weighted mean of k_1 and k_2, calculated based on the fraction of each species before binding. Thus, since k_1 must be $>>k_2$, the k_{on}^{app} value will be very similar to k_1, the on-rate constant for the major species. The experimentally observed K_d value ($k_{off}^{app}/k_{on}^{app}$) will be similar to the one for the major species (as well as the overall K_d). This means that the $\Phi_{binding}$ value from the model in scheme 4 will report on the transition state for the productive pathway leading to the major bound species.

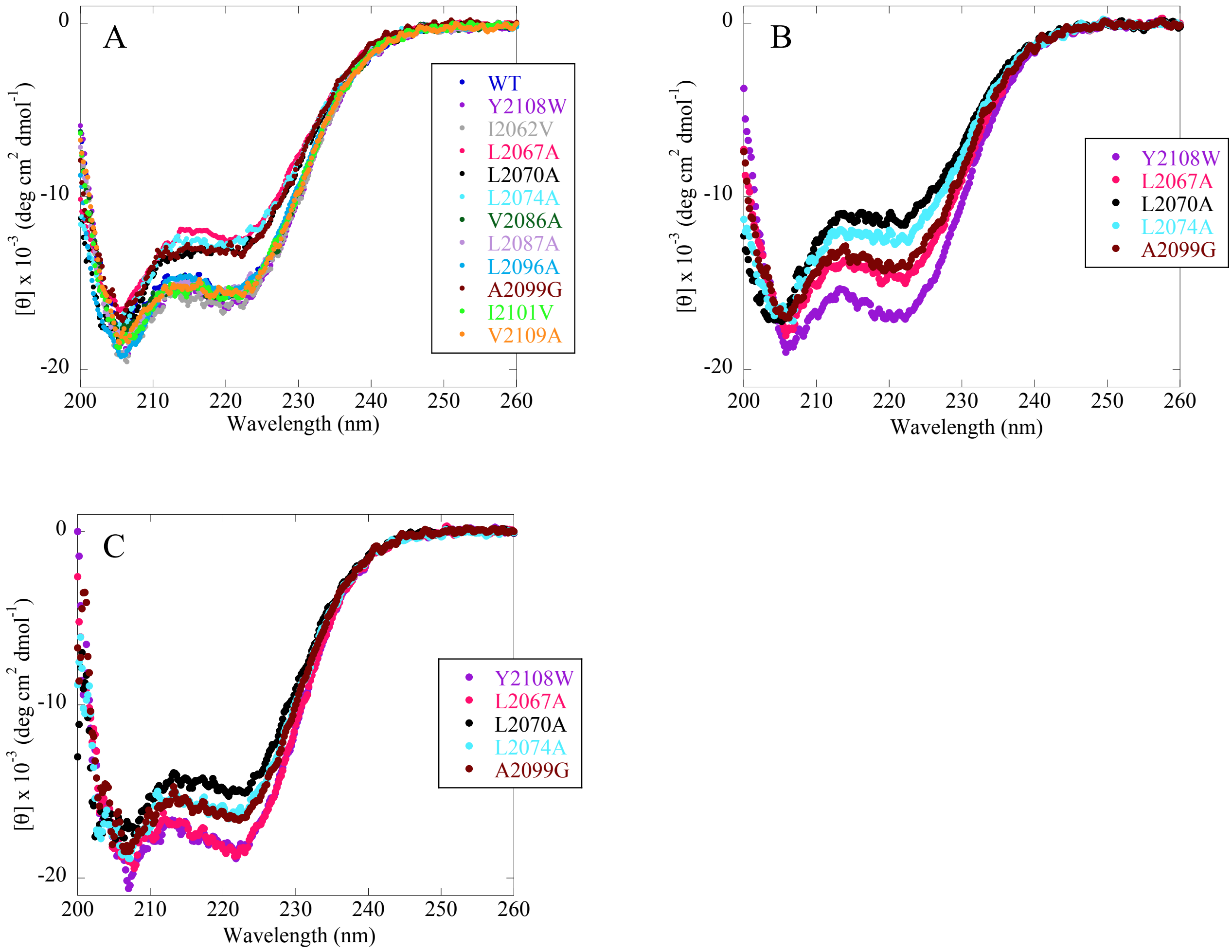

Figure S1. Far-UV circular dichroism experiments of point mutants of NCBD in 20 mM phosphate (pH=7.4), 150 mM NaCl unless otherwise stated at A) 298 K B) mutants with reduced CD signal compared to $NCBD_{Y2108W}$, recorded at 277 K, and C) same mutants as in B) but in buffer supplemented with 0.7 M TMAO and at 277 K.

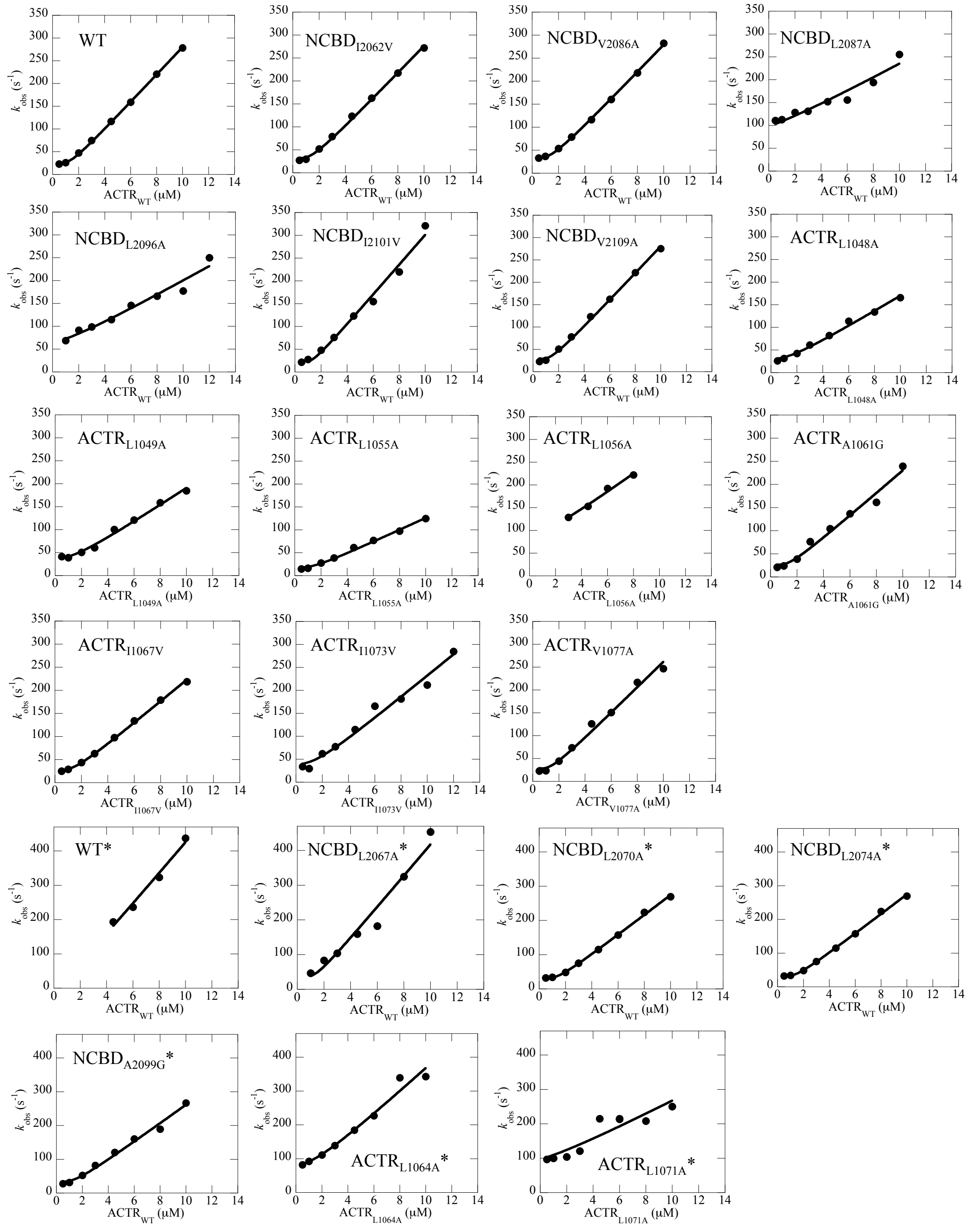

Figure S2. Binding kinetics for all mutants of NCBD$_{Y2108W}$ and ACTR$_{WT}$. WT represents the binding kinetics of NCBD$_{Y2108W}$/ACTR$_{WT}$. An asterisk means that the measurements were performed in 20 mM phosphate (pH=7.4), 150 mM NaCl, 0.7 M TMAO.

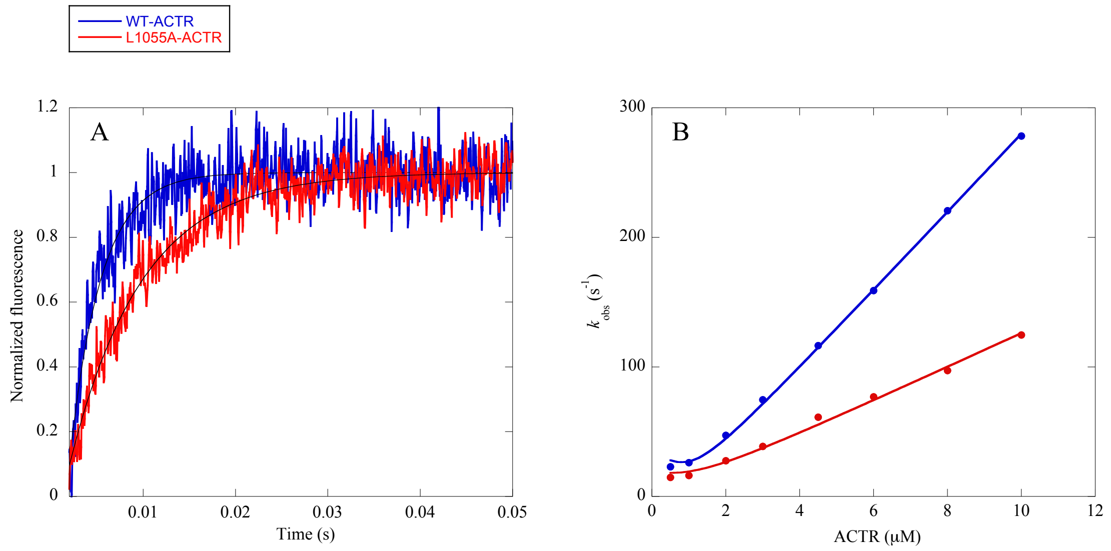

Figure S3. A) Comparison of stopped-flow binding traces between $NCBD_{Y2108W}$/L1055A-ACTR and $NCBD_{Y2108W}$/WT-ACTR. Protein concentrations were 1 μM $NCBD_{Y2108W}$ and 10 μM ACTR. B) k_{obs} plotted against ACTR concentration at 277 K for $NCBD_{Y2108W}$/L1055A-ACTR (red) and $NCBD_{Y2108W}$/WT-ACTR (blue).

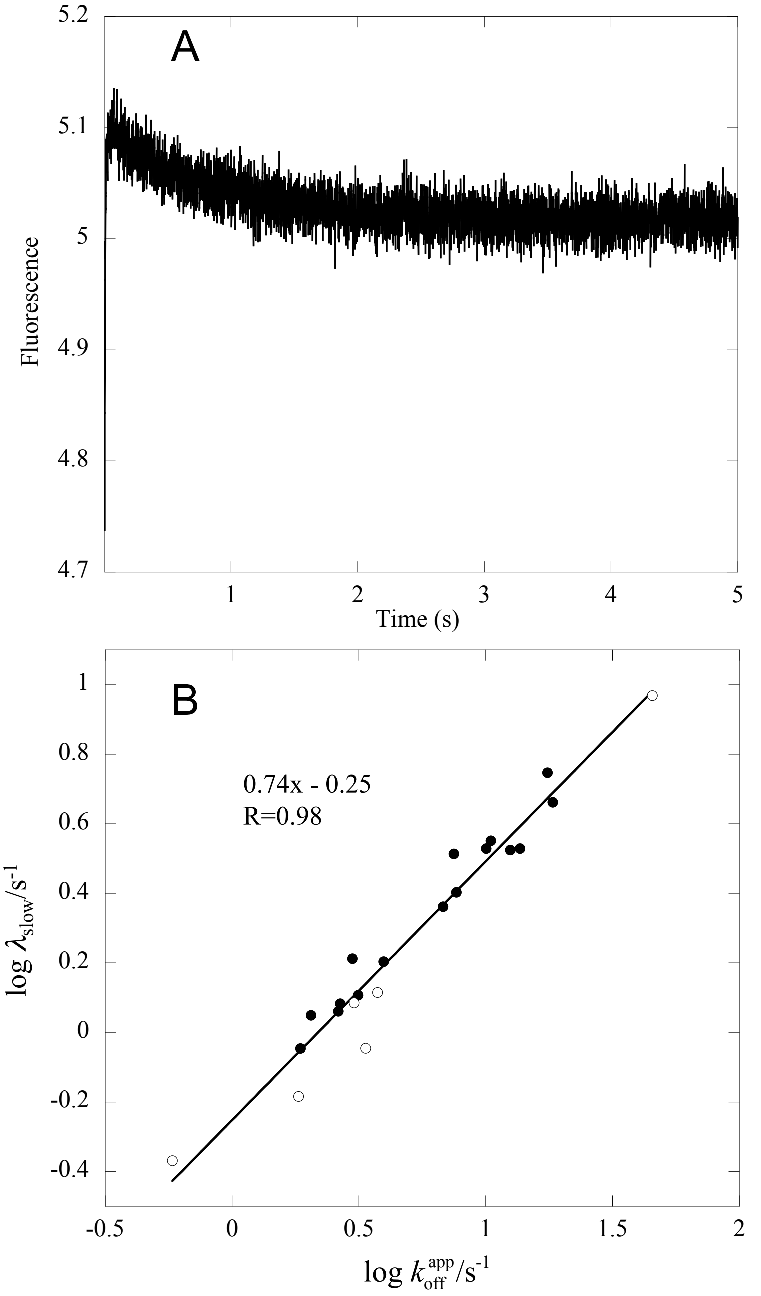

Figure S4. A) Example of a stopped-flow binding trace showing the slow phase. Shown here is the binding between 1 μM I2101V-NCBD and 10 μM WT-ACTR. B) Linear free energy relationship between λ_{slow} and k_{off}^{app} upon mutation. Data that were obtained in 20 mM phosphate (pH=7.4), 150 mM NaCl, 0.7 M TMAO, are shown as open circles.

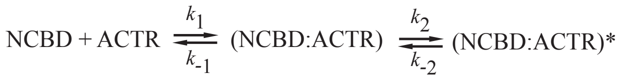

Scheme 1

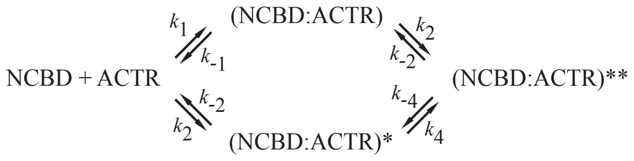

Scheme 2

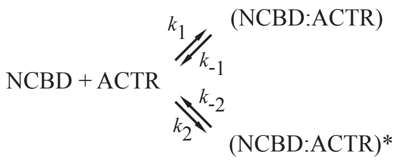

Scheme 3

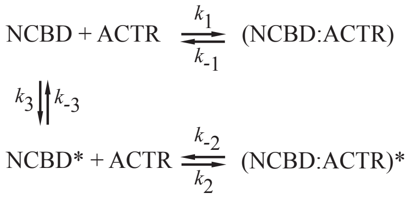

Scheme 4

Figure S5. Four different reaction schemes that may accommodate the experimental data. The stars denote distinct isoforms but should not be compared between schemes. See Supplementary Information text for a discussion on each scheme.

Table S1. Rate constants for the interaction between wild type and mutants of $ACTR_{WT}$ and $NCBD_{Y2108W}$, respectively. Data were collected at 277 K and 20 mM phosphate (pH=7.4), 150 mM NaCl, with or without 0.7 M TMAO, as indicated.

	$ACTR_{WT}$				$NCBD_{Y2108W}$		
$NCBD_{Y2108W}$ mutant	k_{on}^{app} $\mu M^{-1}s^{-1}$	k_{off}^{app} s^{-1}	K_d μM	ACTR mutant	k_{on}^{app} $\mu M^{-1}s^{-1}$	k_{off}^{app} s^{-1}	K_d μM
wild type	28.2 ± 0.6	2.62 ± 0.01	0.093 ± 0.002				
wild type[a]	45.2 ± 3.4	0.58 ± 0.03	0.013 ± 0.001				
I2062V	29.5 ± 0.5	3.96 ± 0.01	0.134 ± 0.002	L1048A	17.0 ± 0.6	10.02 ± 0.02	0.59 ± 0.02
L2067A[a]	45 ± 3	1.83 ± 0.02	0.040 ± 0.003	L1049A	18.7 ± 0.7	18.4 ± 0.3	0.98 ± 0.04
L2070A[a]	29.2 ± 0.9	3.4 ± 0.2	0.12 ± 0.01	L1055A	13.9 ± 0.4	3.0 ± 0.3	0.21 ± 0.02
L2074A[a]	29.3 ± 0.4	3.74 ± 0.04	0.128 ± 0.002	L1056A	21.4 ± 1.7	88 ± 7	4.1 ± 0.5
V2086A	29.7 ± 0.2	10.4 ± 0.2	0.350 ± 0.007	A1061G[c]	24.6 ± 1.2	2.67 ± 0.25<br>1.25 ± 0.05	0.11 ± 0.01
L2087A	15.8 ± 1.8	82 ± 9 [b]	5.2 ± 0.8	L1064A[a]	34.9 ± 2.3	45 ± 9 [b]	1.3 ± 0.3
L2096A	16.2 ± 1.4	44 ± 6	2.7 ± 0.4	I1067V	23.6 ± 0.3	6.8 ± 0.1	0.29 ± 0.01
A2099G[a]	28 ± 1	3.03 ± 0.04	0.110 ± 0.004	L1071A[a]	20 ± 4	77 ± 16 [b]	3.8 ± 1.1
I2101V	33 ± 1	3.14 ± 0.03	0.095 ± 0.003	I1073V[c]	23.5 ± 1.2	7.5 ± 0.2<br>2.8 ± 0.1	0.32 ± 0.02
V2109A	30.2 ± 0.5	2.05 ± 0.02	0.068 ± 0.001	V1077A	28 ± 1	1.86 ± 0.01	0.066 ± 0.003

[a]Measured in 20 mM phosphate (pH=7.4), 150 mM NaCl, 0.7 M TMAO

[b]Determined from fitting to Eq. 1 in main text.

[c]Two dissociation phases were observed.

Table S2. Values for the slow step in the interaction between wild type and mutants of $ACTR_{WT}$ and $NCBD_{Y2108W}$, respectively.

$ACTR_{WT}$		$NCBD_{Y2108W}$	
$NCBD_{Y2108W}$ mutant	λ_{slow} (s^{-1})	$ACTR_{WT}$ mutant	λ_{slow} (s^{-1})
wild type	1.15 ± 0.07		
wild type[a]	0.43 ± 0.03		
I2062V	1.6 ± 0.1	L1048A	3.4 ± 0.3
L2067A	2.5 ± 0.1	L1049A	4.6 ± 0.4
L2067A[a]	0.66 ± 0.02		
L2070A	3.4 ± 0.3	L1055A	1.6 ± 0.1
L2070A[a]	0.9 ± 0.2		
L2074A	3.4 ± 0.2	L1056A	n.v.[b]
L2074A[a]	1.30 ± 0.04		
V2086A	3.6 ± 0.2	A1061G	1.25 ± 0.05
L2087A	n.v.[b]	L1064A[a]	9.3 ± 1.3
L2096A	n.v.[b]	I1067V	2.3 ± 0.1
A2099G	5.6 ± 0.3	L1071A[a]	n.v.[b]
A2099G[a]	1.22 ± 0.02		
I2101V	1.3 ± 0.2	I1073V	3.3 ± 0.5
V2109A	1.1 ± 0.1	V1077A	0.9 ± 0.1

[a]Measured in 20 mM phosphate (pH=7.4), 150 mM NaCl, 0.7 M TMAO

[b]Not visible.

REFERENCES

1. Demarest, S.J. et al. Mutual synergistic folding in recruitment of CBP/p300 by p160 nuclear receptor coactivators. *Nature* **415**, 549-553 (2002).
2. Zhang, W., Ganguly, D. & Chen, J. Residual structures, conformational fluctuations, and electrostatic interactions in the synergistic folding of two intrinsically disordered proteins. *PLoS Comp. Biol.* **8**, e1002353 (2012).
3. Fersht, A.R. & Sato, S. Phi-value analysis and the nature of protein-folding transition states. *Proc. Natl. Acad. Sci. USA* **101**, 7976-81 (2004).
4. Kjaergaard, M., Poulsen, F.M. & Teilum, K. Is a malleable protein necessarily highly dynamic? The hydrophobic core of the nuclear coactivator binding domain is well ordered. *Biophys. J.* **102**, 1627-1635 (2012).
5. Kjaergaard, M., Andersen, L., Nielsen, L.D. & Teilum, K. A Folded Excited State of Ligand-Free Nuclear Coactivator Binding Domain (NCBD) Underlies Plasticity in Ligand Recognition. *Biochemistry* **52,** 1686-1693 (2013).
6. Japrung, D. et al. Single-molecule studies of intrinsically disordered proteins using solid-state nanopores. *Anal. Chem.* **85**, 2449-2456 (2013).
